# Early Prediction of Soybean Traits through Color and Texture Features of Canopy RGB Imagery

**DOI:** 10.1038/s41598-019-50480-x

**Published:** 2019-10-01

**Authors:** Wenan Yuan, Nuwan Kumara Wijewardane, Shawn Jenkins, Geng Bai, Yufeng Ge, George L. Graef

**Affiliations:** 1 Biological Systems Engineering Department, University of Nebraska–Lincoln, Lincoln, NE 68583 USA; 2 Department of Agronomy and Horticulture, University of Nebraska–Lincoln, Lincoln, NE 68583 USA

**Keywords:** Image processing, Computational models

## Abstract

Global crop production is facing the challenge of a high projected demand, while the yields of major crops are not increasing at sufficient speeds. Crop breeding is an important way to boost crop productivity, however its improvement rate is partially hindered by the long crop generation cycles. If end-season crop traits such as yield can be predicted through early-season phenotypic measurements, crop selection can potentially be made before a full crop generation cycle finishes. This study explored the possibility of predicting soybean end-season traits through the color and texture features of early-season canopy images. Six thousand three hundred and eighty-three images were captured at V4/V5 growth stage over 6039 soybean plots growing at four locations. One hundred and forty color features and 315 gray-level co-occurrence matrix-based texture features were derived from each image. Another two variables were also introduced to account for location and timing differences between the images. Five regression and five classification techniques were explored. Best results were obtained using all 457 predictor variables, with Cubist as the regression technique and Random Forests as the classification technique. Yield (RMSE = 9.82, R^2^ = 0.68), Maturity (RMSE = 3.70, R^2^ = 0.76) and Seed Size (RMSE = 1.63, R^2^ = 0.53) were identified as potential soybean traits that might be early predictable.

## Introduction

Increasing population, growing meat and dairy consumption and rising biofuel usage are the key factors for the climbing global demand for crop production^1,2^. By 2050, a 60 to 110% increase in world’s agricultural production may be needed to meet the projected demand^1,3^, which is known as the 2050 challenge. A 2013 study^1^ found that, globally, the average increase rates of yield from 1961 to 2008 for four major crops—maize, rice, wheat and soybean, were far below the adequate levels to meet future demands. Doubts even exist for our ability to maintain current crop yields in the context of a rapidly changing global environment^4^. More land clearing for agriculture and improving the productivity of existing cropland are two solutions for the challenge^3^, however the latter solution is preferred^1^.

Crop productivity can be improved through crop breeding and advanced management practices. Crop breeding aims to improve crop genetic makeup for more desirable traits such as higher yield, however the improvement rate of modern crop breeding in terms of genetic gain is insufficient for the 2050 challenge^5^. Partially, this slow improvement rate is due to the long crop generation cycles^6^. Newly emerged methods such as “speed breeding”, which utilizes prolonged photoperiods, can increase the generation cycles of certain crops in greenhouse from 2–3 to 4–6 per year^6^. However, a greenhouse cannot fully mimic field conditions, plus it has limited space and high running and maintenance costs. In order to select the crop genotypes that are suitable for extensive agricultural production, breeding in field is crucial due to its advantages over breeding in greenhouse. Since field environment cannot be easily altered by humans, the concept of “speed breeding” cannot be realized in field in the same way as if in greenhouse, and alternative methods are needed for accelerating crop breeding research.

The phenotype of a plant results from the interaction between its genotype and environment, and it reflects plant performance under a certain environment. Since the genotype of a plant does not change throughout the course of growth, relationships might exist between plant phenotypes at different time points. If plant traits at the end of a season such as yield can be predicted by plant phenotyping at early-season, breeders then do not have to wait for a full crop generation cycle to make plant selections, thus the speed of crop breeding can be improved. Attempts for early prediction of plant traits have been made in previous research. For example, predicting soybean yield using normalized difference vegetation index (NDVI) measured at reproductive stages^7^; predicting sugar and fiber contents of sugarcane at maturity using the corresponding values measured months before the harvest^8^; predicting leaf nitrogen concentration of almond in summer using leaf nitrogen and boron concentrations in spring^9^; predicting grapevine yield using the number of berries detected at fruit development stages^10^.

To select a phenotyping method that is suitable for large-scale crop breeding research, it needs to be non-destructive and efficient. Advanced instruments such as light detection and ranging (LiDAR) or hyperspectral camera can provide rich information about a plant, however they are typically expensive and can be difficult for people with non-engineering backgrounds to use. Red-green-blue (RGB) cameras, on the other hand, have been widely and long employed in agricultural research. They are cheap and user-friendly, and modern models are able to capture images in high spatial resolutions. With the popularization of smartphones, RGB cameras also have high accessibility. Many well-developed image processing and analysis techniques allow various features from RGB images to be extracted and analyzed, however few have been studied for crop trait early prediction purpose.

Color and texture are two important aspects in digital imagery. Color is the characteristic perceived by human visual system. The color of a plant is closely related with plant physiology. In an image, the color information of a plant can be used for, for example, plant segmentation^11^, plant stress assessment^12^, disease spot detection^13^, or estimating plant traits such as ground cover^14^, biomass^15^, leaf chlorophyll content^16^ and leaf nitrogen concentration^17^. Many vegetation indices based on RGB bands have been developed and studied for accomplishing those tasks. Texture, though lacking a formal definition, is a visual pattern consisting of entities with certain characteristics in terms of color, shape, size, etc. The properties of the entities give the perceived coarseness, smoothness, randomness, uniformity, etc., which are eventually regarded as texture^18^. The essence of texture in digital imagery is the spatial arrangement of pixels with various gray levels^19^. Texture analysis is important in many areas such as remote sensing and medical imaging, and its common applications include image segmentation, image classification and pattern recognition^19^. Although various texture analysis techniques exist, texture features derived from gray-level co-occurrence matrix (GLCM) are the most popular because of their simplicity and adaptability^20^. Interestingly, the value of texture information of RGB image transformations such as vegetation index images has never been investigated to the authors’ knowledge.

The goal of this study was to explore the possibility of soybean trait early prediction using color and texture features of canopy RGB imagery. More specifically, the objectives of the study were:

1. Select the modelling techniques that would provide the best prediction results among the compared ones;
2. Determine which type of variable combination would provide the best prediction results, such as using only color indices, using only texture indices, using both color and texture indices, etc.;
3. Investigate whether the color and texture information of theoretical and empirical transformations of RGB images, namely images in alternative color spaces and vegetation index images based on RGB bands, could improve prediction results;
4. Identify which end-season soybean traits might be predictable through the color and texture features of early-season canopy RGB images.

## GLCM Review

GLCM, originally called gray-tone spatial-dependence matrix, was first introduced by Haralick *et al*. in^21^. It describes the joint probability of pixel pairs at any gray levels, thus is able to represent the texture of an image statistically. GLCM-based texture features have many applications in agricultural research, and some examples are listed in Table 1.

**Table 1:** Examples of agriculture-related research utilizing GLCM-based texture features.

Statistical Approach	Application	Case Study	Reference
Classification	Plant identification	Plant leaf identification using Flavia dataset (32 types of plants) and Foliage dataset (60 types of plants)	^28^
		Identification of grape, mango, chili, wheat, beans and sunflower affected by powdery mildew disease	^47^
		Identification of five *Ficus deltoidea* varieties	^48^
		Recognition of 31 classes of plant leaves	^49^
	Flower identification	Classification of 18 types of flowers	^50^
	Seed identification	Classification for individual kernels of wheat, barley, oats, and rye	^51^
		Classification of wheat and barley kernels	^52^
		Identify four geographical origins of *Jatropha curcas* L. seeds	^53^
		Detection of freefalling wheat kernel damage	^54^
	Pollen identification	Identify ten types of pollen grains in honey	^55^
	Disease identification	Classify lesions of three Phalaenopsis seedling diseases and uninfected leaves	^56^
		Classify diseased wheat leaves at five severity stages	^57^
		Classify healthy, early blight and late blight diseased tomato leaves	^58^
		Classify early blight diseased eggplant leaves and heathy leaves	^59^
		Identify two types of diseased grapevine leaves	^60^
	Stress detection	Detection of three levels of drought stress in maize	^61^
	Weed detection	Identify wild blueberry, weeds and bare spots in field	^62^
		Detection of weeds in rice fields	^63^
		Classify vegetables and weeds in filed	^64^
	Plant mapping	Classification for corn, wheat, soya, pasture, and alfalfa using multipolarization radar data	^65^
		Map invasive *Leucaena leucocephala* using QuickBird satellite imagery	^66^
		Map invasive *Fallopia japonica* using orthophotos	^67^
	Growth stage identification	Phenological stage classification of wheat, barely, lentil, cotton, pepper and corn	^68^
Regression	Trait estimation	Improve the empirical relationship between leaf area index (LAI) and normalized difference vegetation index (NDVI) of forest	^69^
		Estimate age, top height, circumference, stand density and basal area of forest	^70^
		Predict textural class, moisture content, leaf area index and leaf water potential of moss	^38^
		Estimate forest biomass	^71^
		Predict glucose, fructose, sucrose and total sugar content of muskmelon	^72^
		Predict moisture content of quince fruits being dried	^73^
		Predict maize leaf moisture content	^74^
		Estimate leaf nitrogen content of winter wheat	^75^
		Count ear number of wheat growing in filed	^76^

A GLCM can be mathematically expressed as *P*(*i*, *j*, *d*, *θ*), where *i* and *j* stand for pixel intensities, or gray levels of two pixels in a pixel pair, *d* stands for pixel displacement, and *θ* stands for scanning direction. Since calculating a GLCM over the full dynamic range of an image can be prohibitive, quantization is a common practice for reducing the number of gray levels in an image. For 8-bit images, which have 256 gray levels, quantization level can be 8, 16 or 32^22^. However, the tradeoff of this accelerated GLCM calculation is a reduction in image information.

**Figure 1:**
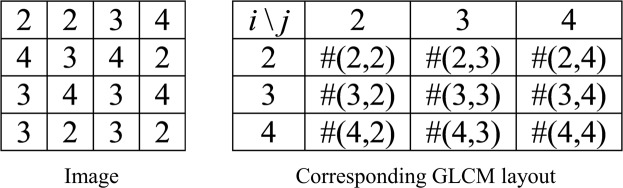
Schematic diagram showing the GLCM layout of an image.

Assume a 4 × 4 image with gray levels specified, then the corresponding GLCM represents the numbers of pixel pairs in the image (Fig. 1).

**Figure 2:**
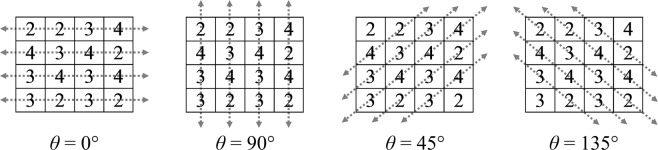
Common scanning directions for generating a GLCM.

To calculate a GLCM, one needs to specify *d* and *θ*. *d* defines the distance between two pixels that can be considered as a “pair”, which is typically set as 1, meaning two adjacent pixels are considered as one pair. *θ* defines the direction along which the pixel pairs lie. 0°, 45°, 90° and 135° are common scanning directions (Fig. 2).

**Figure 3:**
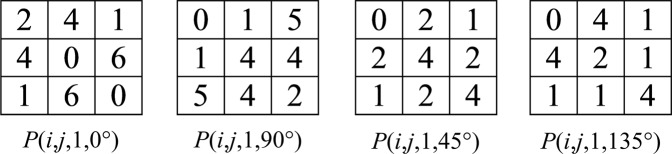
Symmetric GLCM examples of the sample image.

The distinction between two opposite scanning directions is typically ignored, such as left to right versus right to left, since the resulting GLCMs are simply the transpose of each other, then symmetric GLCMs can be employed as shown in Fig. 3^23^, where both directions are considered.

Before extracting texture features, a GLCM needs to be normalized. *p*(*i*, *j*, *d*, *θ*) denotes the normalized GLCM, where:

1 $$p(i,j,d,\theta )=\,\frac{P(i,j,d,\theta )}{{\sum }_{i,j}P(i,j,d,\theta )}$$

as shown in Fig. 4.

**Figure 4:**
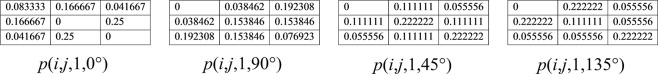
Normalized GLCM examples of the sample image.

Texture features extracted from different GLCMs of the same image can be either averaged or treated as independent variables, though Haralick *et al*. suggested to use the averages^21^.

## Materials and Methods

### Data collection

Soybean canopy images were collected in 2016 over plots growing at four locations using a multi-sensor phenotyping system^24^, which was equipped with C920-C Webcams (Logitech, Lausanne, Switzerland). Soybean plots belonged to 35 yield evaluation experiments in University of Nebraska soybean breeding programs, within which the soybean populations were developed for different purposes, such as improved yield, improved genotype diversity, improved response to water, and improved seed quality metrics. In total 6383 images were captured over 6039 unique plots with measurements repeated for some plots. Among all plots, 2551 unique genotypes exist. Details regarding data collection are listed in Table 2. Images were stored as 8-bit png files with a 2304 × 1536 resolution.

**Table 2:** Soybean plot and data collection details.

Location	Date Planted	Date Harvested	Date Measured	Growth Stage at Measuring	Number of Images
Clay Center, NE	5/20/2016	10/20/2016	6/21/2016	V4/V5	1254
Cotesfield, NE	5/21/2016	10/2/2016	6/23&24/2016	V4/V5	1332
Mead, NE	6/3/2016	10/16/2016	7/6&8/2016	V4/V5	2555
Wymore, NE	6/4/2016	10/31/2016	7/10/2016	V4/V5	1242

### Ground truths

Nine soybean traits were selected for this study, which are defined as the following:

- Yield: seed volume in bushels per acre, adjusted to 13% moisture content, after the seeds have been dried to a uniform moisture content.
- Maturity: the number of days in between the planting date and the date when 95% of the pods have reached their mature color. Delayed leaf drop and green stems are not considered in assigning maturity.
- Height: the average length from ground to the tip of the main stem at maturity, measured in inches.
- Seed Size: seed weight in grams per 100 seeds.
- Protein, Oil, and Fiber: seed composition information was obtained through an Infratec™ 1241 Grain Analyzer (FOSS, Hillerød, Denmark) with a transmittance scanning monochromator spectrometer. Reflectance values were transformed through SB201301 soybean bulk seed and SB201304 soybean sample transport module calibrations provided by the Iowa Grain Quality Laboratory, Iowa State University^25^ to output protein, oil and fiber compositions by weight adjusted to 13% moisture. Ten subsamples were used analyzing plot seed samples, and values were reported as the ten-subsample average.
- Lodging: rated at maturity according to the following scores:

◦ 1: most plants erect.

◦ 2: all plants leaning slightly or a few plants down.

◦ 3: all plants leaning moderately, or 25 to 50% down.

◦ 4: all plants leaning considerably, or 50 to 80% down.

◦ 5: most plants down.

- Seed Quality: rated according to the following scores considering the amount and degree of wrinkling, defective seed coat (growth cracks), greenishness, and moldy or other pigment:

◦ 1: very good.

◦ 2: good.

◦ 3: fair.

◦ 4: poor.

◦ 5: very poor.

Not all ground truths were available for every plot measured. Table 3 shows the availability of each ground truth. Relationships between the soybean traits can be found in Supplementary Information.

**Table 3:** The number of images having the corresponding ground truth available.

Ground Truth	Number of Images
Yield	6001
Maturity	4719
Height	3118
Seed Size	2372
Protein	2801
Oil	2801
Fiber	2801
Lodging	4719
Seed Quality	1866

### Image processing

Image processing was completed using MATLAB R2018b (The MathWorks, Inc., Natick, MA, USA).

#### Pre-processing

For the purpose of enhancing contrast and improving color consistency across images, the contrast of raw images were stretched by saturating the bottom 1% and the top 1% of all pixel values in R, G and B channels respectively. Assume a grayscale image *I*(*x*, *y*), where *x* stands for pixel row position, and *y* stands for pixel column position. In our case, *x* and *y* ranged from 1 to 1536 and 1 to 2304. Then the contrast-enhanced image *E*(*x*, *y*) would be:

2 $$E(x,y)=\{\begin{array}{c}{L}_{N},I(x,y) < {L}_{O}\\ \frac{(I(x,y)-{L}_{O})({U}_{N}-{L}_{N})}{{U}_{O}-{L}_{O}}+{L}_{N},{L}_{O}\ll I(x,y)\ll {U}_{O}\\ {U}_{N},I(x,y) > {U}_{O}\end{array}$$

where *L*_*O*_ and *U*_*O*_ are the original lower and upper limits, which are the 1^st^ and 99^th^ percentile of all pixel values in *I*(*x*, *y*), and *L*_*N*_ and *U*_*N*_ are the new limits, which are 0 and 255 for 8-bit images.

Next, soil background was removed since it contained irrelevant information. It was challenging to segment plants under different lighting and shadowing conditions using one regular thresholding technique. Here we proposed a new plant segmentation method utilizing multiple vegetation indices to maximize segmentation accuracy.

First three vegetation index images were calculated from each contrast-enhanced RGB image: excess green (ExG)^26^, modified excess green (MExG) and color index of vegetation extraction (CIVE), where:

3 $$ExG=\{\begin{array}{l}-1,\,R=G=B=0\\ \frac{2G-R-B}{R+G+B},else\end{array}$$

4 $$MExG=1.262G-0.884R-0.311B$$

5 $$CIVE=0.441R-0.811G+0.385B+18.78745$$

Each of the three vegetation index images was then rescaled to the range of 0 to 1 respectively. The difference image between MExG and CIVE was computed to further enhance the intensity difference between plant pixels and background pixels, then a binary mask *M1*(*x*, *y*) was generated using Otsu’s thresholding technique^27^. A 0.5 threshold was applied to ExG to generate another binary mask *M2*(*x*, *y*). Two masks were overlaid to create the final mask *M*(*x*, *y*) where:

6 $$M(x,y)=\{\begin{array}{l}NA,M1(x,y)=M2(x,y)=0\\ 1,else\end{array}$$

**Figure 5:**
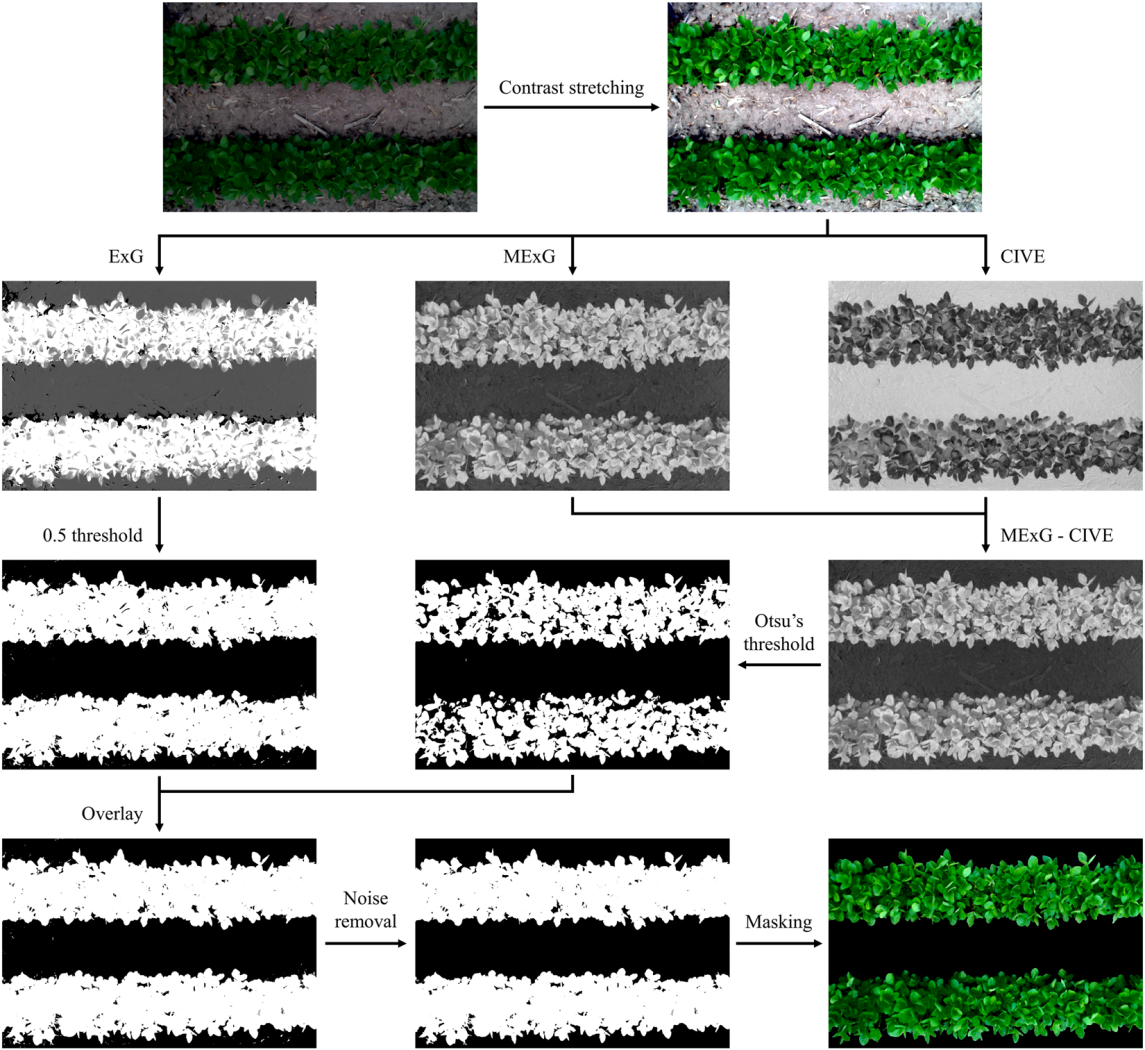
Flowchart of image pre-processing.

Instead of using zero, NA values were adopted here to avoid the influence of a large number of zero in a masked image when computing color and texture features. The noise of *M*(*x*, *y*) was cleaned by removing objects with 300 or fewer connected pixels. To this point *M*(*x*, *y*) was ready to be used for removing soil background from any images calculated later (Fig. 5).

#### Image transformation

Four common color spaces, and 20 vegetation indices based on RGB bands were selected to represent the theoretical and empirical transformations of an RGB image (Table 4). Plus the original RGB color space, in total (1 + 4) × 3 + 20 = 35 transformed images were calculated from each contrast-enhanced RGB image, then mask *M*(*x*, *y*) was applied to all transformed images.

**Table 4:** List of theoretical and empirical RGB image transformations.

Type	Name	Abbreviation	Description	Note	Reference
Original	Red	R	R channel from RGB color space	Raw values were adjusted by contrast stretching. Values ranged from 0 to 255.	^17^
	Green	G	G channel from RGB color space		
	Blue	B	B channel from RGB color space		
Theoretical transformation	X	X	X channel from CIE 1931 XYZ color space	CIE 1931 2° Standard Observer; CIE Standard Illuminant D65	^77^
	Y	Y	Y channel from CIE 1931 XYZ color space		
	Z	Z	Z channel from CIE 1931 XYZ color space		
	L-star	L^*^	L^*^ channel from CIE 1976 L^*^a^*^b^*^ color space	CIE Standard Illuminant D65	^17^
	a-star	a^*^	a^*^ channel from CIE 1976 L^*^a^*^b^*^ color space		
	b-star	b^*^	b^*^ channel from CIE 1976 L^*^a^*^b^*^ color space		
	Hue	H	H channel from HSI color space		^17, 78^
	Saturation	S	S channel from HSI color space		
	Intensity	I	I channel from HSI color space		
	Y-prime	Y’	Y’ channel from Y’CbCr color space		^79^
	Cb	Cb	Cb channel from Y’CbCr color space		
	Cr	Cr	Cr channel from Y’CbCr color space		
Empirical transformation	Normalized red	NR	${\rm{NR}}=\frac{{\rm{R}}}{{\rm{R}}+{\rm{G}}+{\rm{B}}}$	Equations simplified. Abbreviations also known as r, g, b.	^26^
	Normalized green	NG	${\rm{NG}}=\frac{{\rm{G}}}{{\rm{R}}+{\rm{G}}+{\rm{B}}}$		
	Normalized blue	NB	${\rm{NB}}=\frac{{\rm{B}}}{{\rm{R}}+{\rm{G}}+{\rm{B}}}$		
	Excess red	ExR	${\rm{ExR}}=\frac{{\rm{1}}{\rm{.4R}}-{\rm{G}}}{{\rm{R}}+{\rm{G}}+{\rm{B}}}$	Equation simplified.	^80^
	Excess blue	ExB	${\rm{ExB}}=\frac{{\rm{1}}{\rm{.4B}}-{\rm{G}}}{{\rm{R}}+{\rm{G}}+{\rm{B}}}$	Equation simplified.	^81^
	Excess green red	ExGR	${\rm{ExGR}}=\frac{{\rm{3G}}-{\rm{2}}{\rm{.4R}}-{\rm{B}}}{{\rm{R}}+{\rm{G}}+{\rm{B}}}$	Equation simplified.	^82^
	Green blue difference	GBD	${\rm{GBD}}={\rm{G}}-{\rm{B}}$		^83^
	Red blue difference	RBD	${\rm{RBD}}={\rm{R}}-{\rm{B}}$		
	Red green difference	RGD	${\rm{RGD}}={\rm{R}}-{\rm{G}}$		
	Green red ratio	GRR	${\rm{GRR}}=\frac{{\rm{G}}}{{\rm{R}}}$		^14, 84^
	Green blue ratio	GBR	${\rm{GBR}}=\frac{{\rm{G}}}{{\rm{B}}}$		^83^
	Normalized green red difference	NGRD	${\rm{NGRD}}=\frac{{\rm{G}}-{\rm{R}}}{{\rm{G}}+{\rm{R}}}$	Also known as normalized difference index (NDI) or green red vegetation index (GRVI).	^11, 15^
	Normalized green blue difference	NGBD	${\rm{NGBD}}=\frac{{\rm{G}}-{\rm{B}}}{{\rm{G}}+{\rm{B}}}$		^84, 85^
	Modified normalized green red difference	MNGRD	${\rm{MNGRD}}=\frac{{{\rm{G}}}^{2}-{{\rm{R}}}^{2}}{{{\rm{G}}}^{2}+{{\rm{R}}}^{2}}$	Also known as modified green red vegetation index (MGRVI).	^86^
	Visible band difference	VD	${\rm{VD}}=\frac{{\rm{2G}}-{\rm{B}}-{\rm{R}}}{{\rm{2G}}+{\rm{B}}+{\rm{R}}}$	Also known as green leaf index (GLI).	^87, 88^
	Red green blue vegetation index	RGBVI	${\rm{RGBVI}}=\frac{{{\rm{G}}}^{2}-{\rm{B}}\times {\rm{R}}}{{{\rm{G}}}^{2}+{\rm{B}}\times {\rm{R}}}$		^86^
	Crust index	CI	${\rm{CI}}=\frac{{\rm{2B}}}{{\rm{R}}+{\rm{B}}}$	Equation simplified.	^83^
	Color index of vegetation extraction	CIVE	${\rm{CIVE}}={\rm{0}}{\rm{.441R}}-{\rm{0}}{\rm{.811G}}+{\rm{0}}{\rm{.385B}}+{\rm{18.78745}}$		^89^
	Triangular greenness index	TGI	${\rm{TGI}}={\rm{95G}}-{\rm{35R}}-{\rm{60B}}$	Equation simplified.	^16^
	Modified excess green	MExG	$\mathrm{MExG}\,=1{\rm{.262G}}-{\rm{0}}{\rm{.884R}}-{\rm{0}}{\rm{.311B}}$		^90^

The famous index ExG was not listed in the table because ExG has a value range of −1 to 2, and when it is normalized to the range of 0 to 1, ExG has an identical expression as NG.

**Figure 6:**
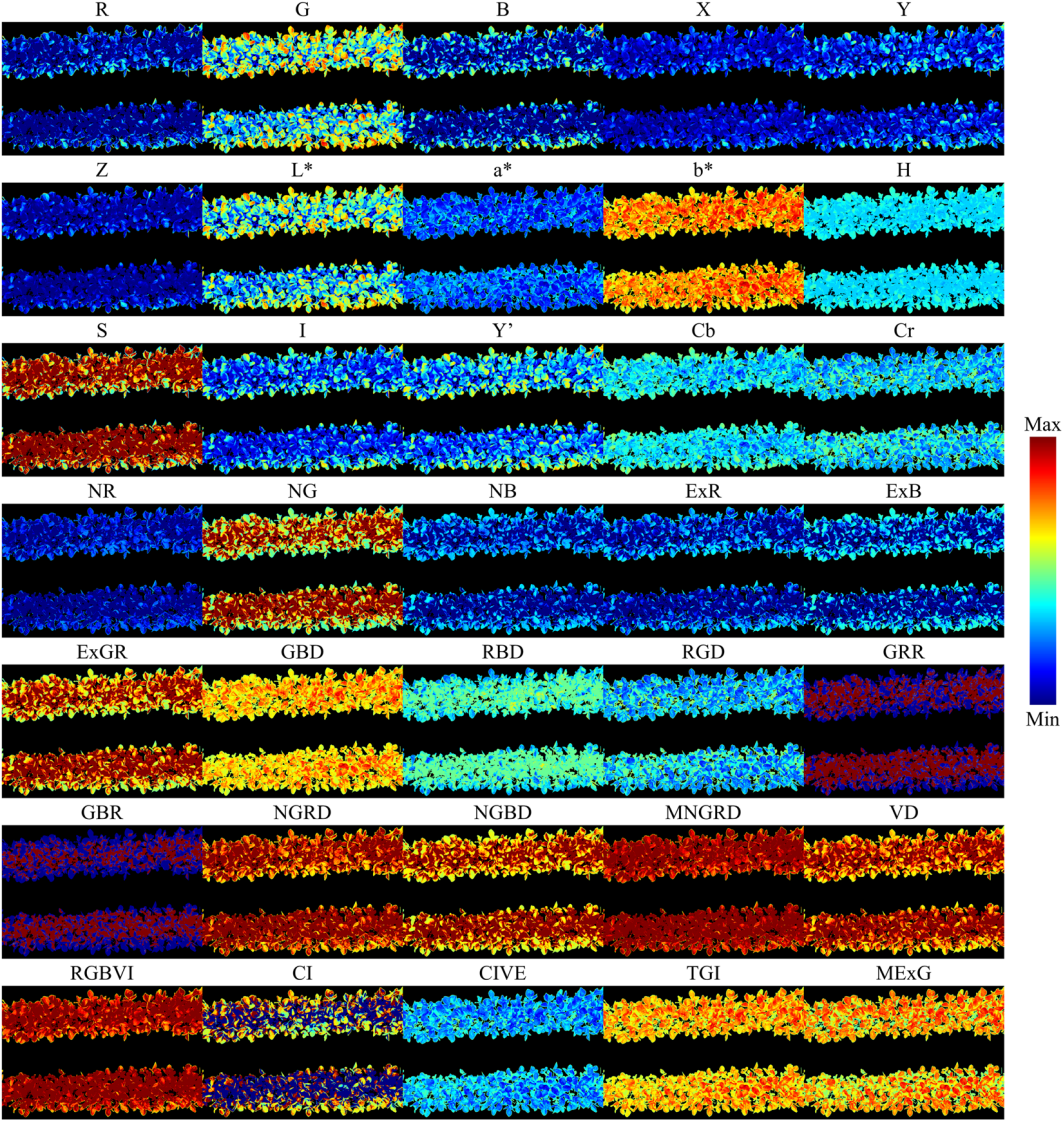
Examples of colorized transformed images containing different color and texture information.

For each of the 35 transformed images, if applicable, non-mask NA values and negative infinity values were replaced as the minimum real value of the image, and positive infinity values were replaced as the maximum real value of the image. All pixel intensity values of transformed images were stored in double format, meaning decimal places were not rounded. Figure 6 shows various texture patterns carried by different transformed images derived from the same RGB image. The images in Fig. 6 were colorized for viewing convenience, and the color scheme corresponded to the value range of an image before mask *M*(*x*, *y*) was applied.

### Image feature extraction

#### Color Features

For each of the 35 transformed images, four color indices were calculated: mean (μ), standard deviation (σ), skewness (θ) and kurtosis (δ)^28^. Since for each soybean plot the cameras were able to capture the majority of the canopy, we assumed the plant pixels in each image followed a population distribution instead of a sample distribution.

Take a transformed image *T*(*x*, *y*) where the number of plant pixels, or non-NA values is N, then:

7 $$\mu =\frac{{\sum }_{x}{\sum }_{y}T(x,y)}{N}$$

8 $$\sigma =\sqrt{\frac{{\sum }_{x}{\sum }_{y}{(T(x,y)-\mu )}^{2}}{N}}$$

9 $$\theta =\frac{{\sum }_{x}{\sum }_{y}{(T(x,y)-\mu )}^{3}}{N{\sigma }^{3}}$$

10 $$\delta =\frac{{\sum }_{x}{\sum }_{y}{(T(x,y)-\mu )}^{4}}{N{\sigma }^{4}}$$

Notice NA values from mask *M*(*x*, *y*) were ignored in the calculations above. In total 35 × 4 = 140 color indices were derived from each original RGB image.

#### Texture features

It is reasonable to assume that the transformed images cannot contain more information than the original RGB images. Before extracting texture features, each of the 35 transformed images without mask *M*(*x*, *y*) applied was first rescaled to 0 to 255 and rounded as integers to reduce computational complexity, then mask *M*(*x*, *y*) was applied. Two symmetric GLCMs *p*(*i*, *j*, 1, 0°) and *p*(*i*, *j*, 1, 90°) were calculated from each transformed image. Notice NA values were ignored when computing GLCMs. Nine texture indices were calculated from each GLCM: maximum probability (MP), mean (MEA), variance (VAR), correlation (COR), angular second moment (ASM), entropy (ENT), dissimilarity (DIS), contrast (CON) and inverse difference moment (IDM)^21,29^, where:

11 $$MP=max(p(i,j,d,\theta ))$$

12 $$MEA={\sum }_{i,j}ip(i,j,d,\theta )={\sum }_{i,j}jp(i,j,d,\theta )$$

13 $$VAR={\sum }_{i,j}{(i-MEA)}^{2}p(i,j,d,\theta )={\sum }_{i,j}{(j-MEA)}^{2}p(i,j,d,\theta )$$

14 $$\begin{array}{rcl}COR & = & {\sum }_{i,j}\frac{(i-MEA)(j-MEA)p(i,j,d,\theta )}{VAR}\\  & = & {\sum }_{i,j}\frac{ijp(i,j,d,\theta )-ME{A}^{2}}{VAR}\end{array}$$

15 $$ASM={\sum }_{i,j}p{(i,j,d,\theta )}^{2}$$

16 $$ENT=-\,{\sum }_{i,j}p(i,j,d,\theta )lo{g}_{2}(p(i,j,d,\theta ))$$

17 $$DIS={\sum }_{i,j}|i-j|p(i,j,d,\theta )$$

18 $$CON={\sum }_{i,j}{(i-j)}^{2}p(i,j,d,\theta )$$

19 $$IDM=\sum _{i,j}\frac{p(i,j,d,\theta )}{1+{(i-j)}^{2}}$$

Confusions exist in the naming and calculation of GLCM-based texture features among literature, and the following are a few clarifications. Eqs 12 and 13 are only valid for a symmetric GLCM. ASM is sometimes named as energy, while energy is sometimes defined as the square root of ASM. Both 2 and Euler’s number e can be used as the base of the logarithm in Eq. 16,also Eq. 16 assumes 0 × log0 = 0. IDM is also called inverse difference, homogeneity or local homogeneity, however the denominator of homogeneity’s expression is sometimes defined as 1 + |i − j|.

After obtaining the same texture features from two GLCMs of the same image, such as MP of *p*(*i*, *j*, 1, 0°) and MP of *p*(*i*, *j*, 1, 90°), two texture indices were averaged as one. In total 35 × 9 = 315 texture indices were derived from each original RGB image.

### Data analysis

The dataset was randomly split into two segments containing 70% and 30% of all data entries for model calibration and validation. Five regression modelling techniques, namely Partial Least Squares Regression (PLS), Random Forests (RF), Cubist (CB), Artificial Neural Networks (ANN) and Support Vector Regression (SVR) were explored to model for Yield, Maturity, Height, Seed Size, Protein, Oil and Fiber. Five classification techniques, namely Partial Least Squares Discriminant Analysis (PLSDA), RF, Linear Discriminant Analysis (LDA), ANN and Support Vector Machines (SVM) were explored to model for Lodging and Seed Quality. All predictor variables were standardized by removing the mean and scaling to unit variance before used for calibrating models. Model tuning was completed through 10 random segment cross-validation. The data analysis was conducted in R language^30^ using package caret^31^, nnet^32^, pls^33^, cubist^34^, randomForests^35^, kernlab^36^ and MASS^32^.

Calibrated models were used to predict for the validation dataset. Prediction statistics, including root mean square error (RMSE), coefficient of determination (R^2^), Bias, Accuracy, and Cohen’s kappa coefficient (Kappa) were calculated to evaluate model performance. RMSE indicates the average prediction error compared to the observations. R^2^ indicates the percentage of observation variance that is explained by the model. Bias indicates the average prediction deviation from the observations. Accuracy indicates the percentage of overall accurate classifications. Kappa indicates the agreement between observed and predicted classes. The statistics were defined as the following:

20 $$RMSE=\sqrt{\frac{1}{n}\sum _{i}{({P}_{i}-{O}_{i})}^{2}}$$

21 $${R}^{2}=1-\frac{{\sum }_{i}{({O}_{i}-{P}_{i})}^{2}}{{\sum }_{i}{({O}_{i}-\bar{O})}^{2}}$$

22 $$Bias=\frac{1}{n}\sum _{i}({P}_{i}-{O}_{i})$$

23 $$Accuracy=\frac{c}{n}$$

24 $$Kappa=\frac{Accuracy-E}{1-E}$$

where *n* is the number of observations or the number of data entries of the validation dataset, *P*_*i*_ is the i^th^ prediction, *O*_*i*_ is the i^th^ observation, $\bar{O}$ is the mean of observations, and *c* is the number of correct classifications. Notice *n* was different for each soybean trait because of the data availability (Table 3). *E* is defined as:

25 $$E=\frac{1}{{n}^{2}}{\sum }_{k}n{p}_{k}n{o}_{k}$$

where *k* is the k^th^ class, *np*_*k*_ is the number of predictions in k^th^ class, *no*_*k*_ is the number of observations in k^th^ class.

For the first objective, all color and texture indices (140 + 315 = 455 variables) were used as predictor variables, and all 10 modelling techniques were employed. Results of different techniques were compared and the best modelling techniques were chosen based on RMSE.

Since the RGB images were captured over different locations at different dates, we introduced another two variables to improve model robustness: Location and Time (LnT). Variable “Location” contained number 1, 2, 3 and 4 representing the four locations where the soybean plots grew. Variable “Time” was the number of days in between the planting date and the measuring date. For the second objective, using the modelling techniques chosen above, four types of variable combinations were investigated: only color indices (140 variables), only texture indices (315 variables), both color and texture indices (455 variables), color and texture indices and LnT (457 variables). The best variable combination was chosen based on RMSE.

Among 35 types of transformed images, only R, G and B could be considered as direct measurements. Therefore, the color and texture features of R, G and B represented the original RGB image, and the rest represented theoretical and empirical transformations of the RGB image (Table 4). To investigate the third objective, using the modelling techniques and the variable combination chosen above, new models were calibrated for all soybean traits, using a combination of color features of R, G and B (3 × 4 = 12 variables), texture features of R, G and B (3 × 9 = 27 variables), or LnT (2 variables).

## Results

Full modelling results can be found in Supplementary Information.

### Objective 1

Prediction results of various techniques were not drastically different in terms of RMSE or Accuracy, however they fluctuated more in terms of R^2^ or Kappa. Comparing the worst results to the best, RMSE would increase by 5 to 42%, on average 16%, and Accuracy would decrease by 9 to 11%, on average 10%, while R^2^ would decrease by 8 to 62%, on average 31%, and Kappa would decrease by 100 to 105%, on average 103%.

Using RMSE or Accuracy of the validation dataset as the standard, CB consistently provided better regression predictions than other techniques except for Fiber, and RF performed the best for classification predictions. We identified CB and RF as the best techniques for the proceeding regression and classification tasks.

### Objective 2

Results showed different variable combinations did not make a big difference in terms of RMSE, R^2^, Accuracy and Kappa for the majority of the soybean traits. Comparing the worst predictions to the best, RMSE would increase by 1 to 12%, on average 7%, R^2^ would decrease by 8 to 42%, on average 15%, Accuracy would decrease by 0.7 to 4%, on average 2%, and Kappa would decrease by 12 to 50%, on average 31%.

Except for Seed Size and Fiber, the variable combination of color, texture and LnT always provided better results, thus we identified it as the best variable combination for both regression and classification and used for the proceeding analysis.

### Objective 3

Since in Objective 2 the combination of color, texture and LnT was identified as the best, new models were calibrated using both color and texture features of R, G and B as well as LnT as predictor variables (12 + 27 + 2 = 41 variables).

Even though using all variables always provided better predictions, comparable results were obtained using color and texture features of only R, G, and B. Comparing the results of two models for each ground truth, for all nine soybean traits, the percentage difference in terms of RMSE, R^2^, Accuracy and Kappa varied in between 0.04 to 7%, 2 to 31%, 1 to 3% and 2 to 50%. Results suggested that the color and texture information of RGB image transformations could only bring marginal improvements to the models calibrated through the color and texture information of original RGB images.

### Objective 4

**Figure 7:**
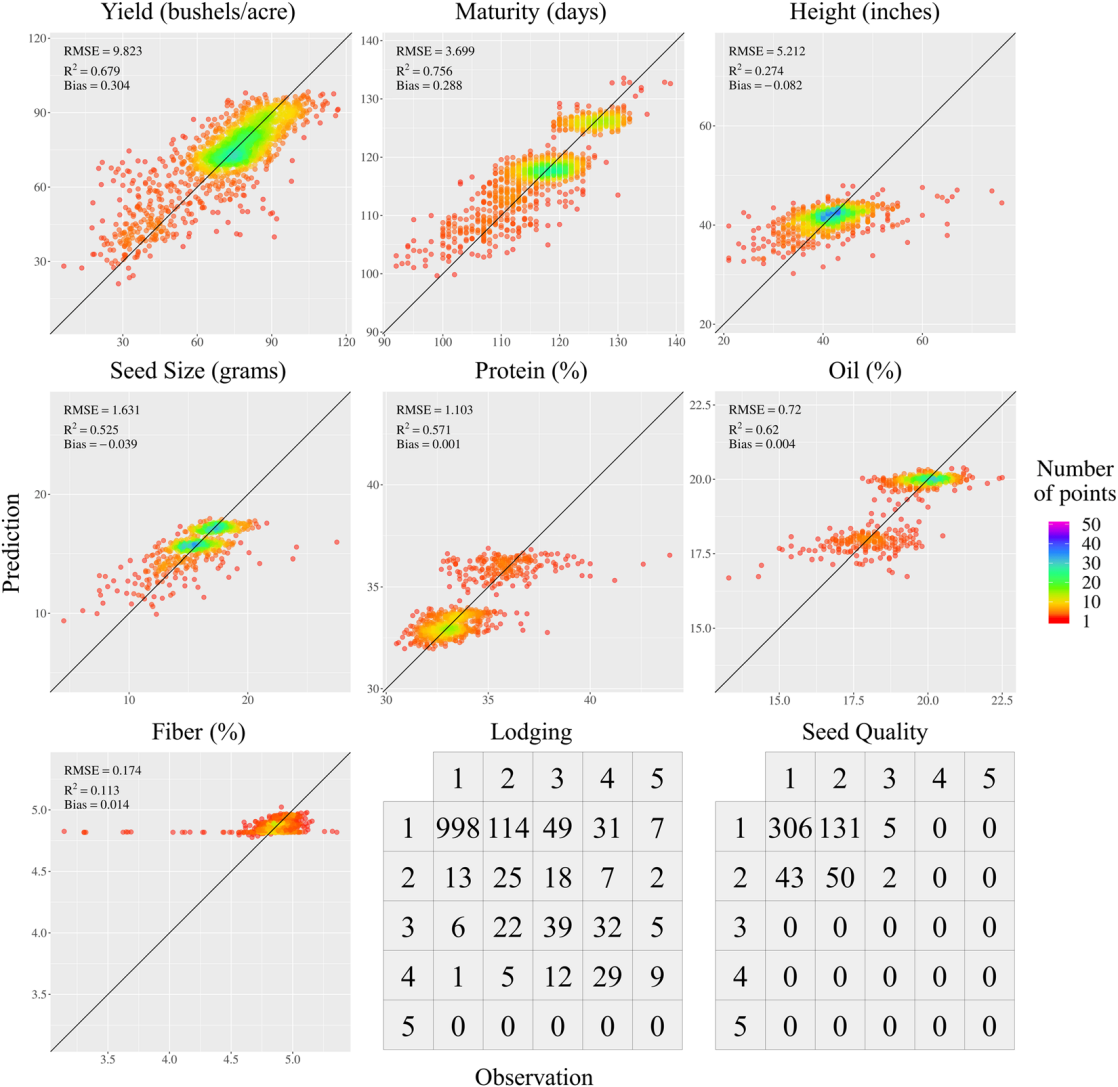
Prediction results for nine soybean traits using all 457 predictor variables.

When using all 457 variables as predictor variables, CB as the regression technique, and RF as the classification technique, prediction results for all soybean traits were presented below (Fig. 7).

Considering the value range of each soybean trait, Seed Size, Protein, Oil and Fiber had small RMSEs, Yield and Maturity had fair RMSEs, and Height had a large RMSE. Yield, Maturity, Seed Size, Protein and Oil all had reasonable R^2^s, whereas Height and Fiber had low R^2^s indicating models were not able to explain large percentages of the data variances. All soybean traits had very small Biases. Both Lodging and Seed Quality had fair Accuracies, however their Kappas were very low. The reason that caused this phenomenon might be the imbalanced data distribution, meaning Lodging and Seed Quality had large proportions of low rating scores, while only a few high rating scores existed. In this scenario even if a model classified all data entries as low rating, Accuracy of the result could still be high.

Data clusters were observed in Maturity, Seed Size, Protein and Oil. When compared to the rest three locations, Clay Center had the highest overall Maturity distribution, and the cluster at the upper-right corner in Maturity represented the soybean plots influenced by Clay Center’s location effect. Similar to Maturity, clusters in Seed Size also indicated location difference. The Seed Size distributions of Cotesfield and Wymore were centered around 17 while Clay Center and Mead were centered around 15, thus each of the two clusters in Seed Size represented two locations. The clusters in Protein and Oil showed a difference in between soybean populations. The cluster at the upper-right corner in Protein and the cluster at the lower-left corner in Oil represented the same soybean population, which was developed for improved genotype diversity. All other soybean populations behaved similarly in Protein and Oil.

Abnormally low values of Fiber existed. Per the consequential inspection of the data, there were 14 potential Fiber outliers if 4.3 was used as the threshold, and the corresponding Protein and Oil values tended to be in the high range (37–43.9) and medium range (18–19.5) respectively. As Protein, Oil and Fiber were measured by the same instrument simultaneously, we eliminated the possibility of instrument malfunctioning and kept all Fiber data entries since the corresponding Protein and Oil values appeared to be in the normal ranges.

Based on the overall consideration of the prediction results, we identified Yield, Maturity and Seed Size as the potential soybean traits that might be early predictable.

## Discussion

### Results of the study

When it comes to different variable combinations, texture alone always provided better predictions than color alone, which implied that texture features might carry more meaningful information than color features. As would be discussed in the next section, complex plant canopy structures can affect the values of texture indices, while color indices can indicate plant overall vigor and health. Unexpectedly, the combination of color and texture did not perform better than color or texture alone in terms of RMSE for five soybean traits, which indicated possible information overlapping in between color and texture features. Since the images were taken at different dates over soybean plots growing at different locations, the soil type and climate difference, as well as the number of days after plant emergence could have a significant impact on plant phenotype, or the canopy appearance in this study. Therefore it was not a surprise that the introduction of LnT provided the best results for seven out of nine soybean traits.

**Figure 8:**
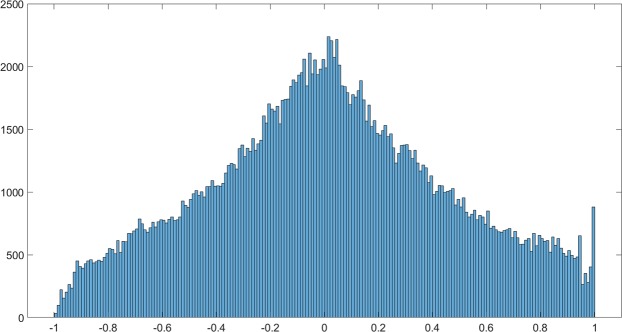
Histogram of correlation coefficients between 455 color and texture indices of 6383 RGB images.

An interesting finding in this study was that the RGB image transformations did not contain much additional valuable information compared to the original RGB images. The models calibrated using only 41 variables provided comparable results to the models calibrated using all 457 variables. Since every set of 35 transformed images were derived from one single RGB image, linear or non-linear relationships existed in between them, thus the color and texture indices of different images might carry similar information. Figure 8 is the histogram of correlation coefficients (455^2^ = 207025) in the correlation matrix between 455 color and texture indices of all 6383 RGB images. As shown in the figure, there was a considerable amount of variables having strong positive or negative correlations with each other, which validated our speculation on information overlapping in between the image indices. There were several reasons why we did not perform any feature selection for our dataset. First, the machine learning techniques that we used in this study do not have any statistical assumptions about the data, also the classical techniques such as PLSR inherently have the ability to handle collinearity^37^, therefore feature selection was not a compulsory step. Secondly, feature selection is commonly used for reducing the computational complexity of model calibration, whereas in our case the model training did not require as many resources. Thirdly, for a dataset when its number of predictors is greater than its number of samples, feature selection is important for preventing overfitting, while this issue did not apply to our dataset. Lastly, since generally the more predictor variables there are the better modelling results tend to be, we decided not to filter out any variables beforehand. Nevertheless, the color and texture features of RGB image transformations marginally improved the modelling results for all soybean traits.

### Agronomical interpretation

The results suggested the possibility of early predicting several end-season soybean traits through the color and texture features of early-season canopy images. Since this subject was rarely explored, the true reasons for this possibility remained mysterious. Ushada *et al*.^38^ estimated moss traits through GLCM-based canopy texture features, and they proposed a black box relationship between canopy parameters and canopy images. In this section a set of arguments are presented in an attempt to rationalize the findings by connecting plant science (e.g. plant parameters) with digital image processing (e.g. color and texture features of canopy images).

Plant developmental traits, such as plant architecture and leaf features, are important factors that determine plant overall performance and can be reflected in an early-season canopy image. Since plant canopy appearance is influenced by such plant parameters, it is logical to assume the color and texture features of a plant canopy image are indicating, or representing certain plant parameters as well as the interactions between them. We identified five major plant parameters below that can be represented by the color and texture information of a canopy image. In other words, the variation of the color and texture indices among different canopy images is mainly caused by the following plant developmental traits:

- Leaf color
  Plant leaf color is associated with biotic and abiotic stresses in plants, such as plant diseases^39^ and nutrient deficiencies^40^, which would typically lead to chlorophyll destruction or chlorophyll formation failure. One common type of tool in crop nitrogen management is a leaf color chart, which utilizes relative leaf greenness as an indicator for leaf nitrogen status. A healthy plant leaf should have a uniform green color distribution, and the corresponding canopy RGB image should have small standard deviations in all three channels. A diseased leaf may have necrotic lesions with non-green colors, which leads to larger standard deviations in all channels because of the nonuniform color distribution. Nutrient deficient or drought-stressed leaves often have chlorosis, which can lead to shifts of means in three channels. Essentially leaf color indicates plant vigor and health, and it is reasonable to imagine vigorous young plants having better performance later on.
- Leaf shape
  Plants with different genotypes can have diverse leaf shapes, which would further influence the efficiency of light harvesting when leaf area density is high. From the perspective of a 2D image, leaf shape is also affected by leaf or branch angle, which has a huge effect on the amount of light that can be received by a leaf. Though not being observed in our images, insect damages, plant diseases or environmental stresses can also change the shape of a leaf. In relation to canopy imagery, texture indices are affected by the shape of leaves since leaves are the fundamental subunits that give the overall canopy texture appearance. Leaf shape contains information regarding plant health and photosynthetic efficiency, thus is partially responsible for plant end-season performance.
- Leaf size
  Since our images were all collected at the same growth stages, the leaf size difference between soybean plots could denote plant growth rates. Also leaf size is directly related with cell number and chlorophyll content, which could determine plant photosynthetic capacity^41^. Both plant growth rate and photosynthetic capacity have been found to be correlated with yield^42,43^. Large leaf size can give plant canopies a “coarse” texture appearance, while small leaf size gives a “fine” look to canopies. This canopy appearance difference would eventually affect the values of texture indices.
- Leaf area density
  Leaf area density describes how close plant leaves distribute spatially. Due to similar reasons for leaf angle and leaf size, leaf area density directly influences plant photosynthetic capacity, also it has an impact on plant photosynthetic efficiency by affecting the quantity of light interception, which in the long term can have a substantial accumulated effect on plant end-season performance. Also leaf area density indirectly shows the number of stems or branches, which is usually negatively correlated with plant height and lodging. High leaf area density can add complexity to plant canopy texture, whereas canopies with lower leaf area densities would have “simpler” appearances.
- Plant density
  As the seeding rates for all soybean plots that we measured were the same, plant density showing in the images indicates the emergence rate and early plant population of a plot. Also plant density interferes with plant photosynthetic efficiency through influencing light interception efficiency. Soybean plots with higher plant density would appear more “uniform”, while the ones with low plant density can have an “irregular” canopy texture. In general one can expect a plot with fewer plants emerged to have less final yield.

**Figure 9:**
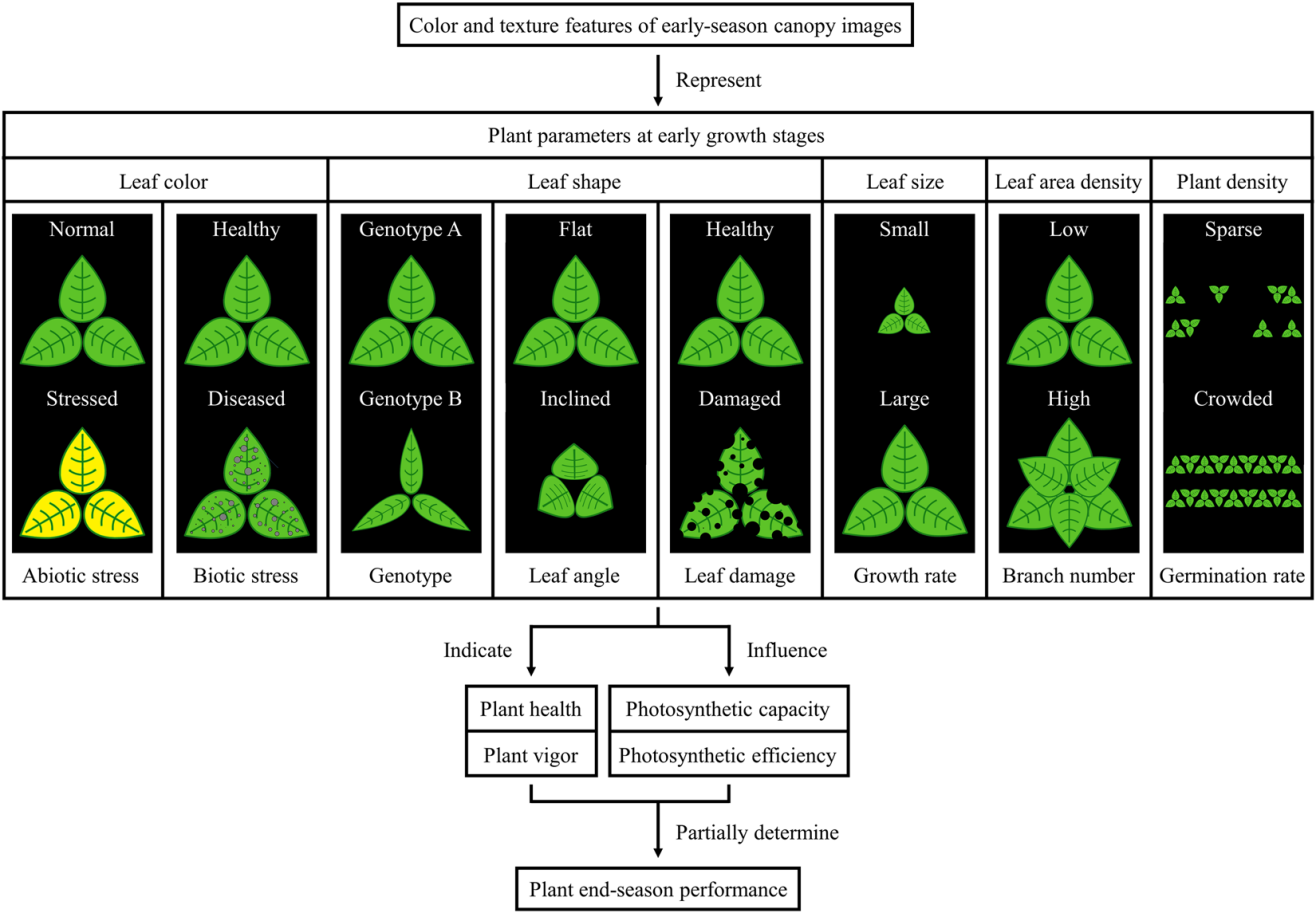
Schematic diagram explaining the potential relationships between color and texture information of early-season canopy images and end-season plant performance.

In summary, as the color and texture indices were statistically derived from early-season canopy images, we speculate that they potentially represent various intertwined characteristics of a plant, such as leaf color, leaf shape, leaf angle, branch angle, leaf size, plant growth rate, leaf area density, stem number, branch number, germination rate, etc. These plant developmental parameters would further indicate or determine plant vigor, plant health, plant drought resistance, plant photosynthetic efficiency, plant photosynthetic capacity, etc. at early growth stages, which can have significant impacts on plant overall performance (Fig. 9).

### Limitations of the study and directions for future studies

An image is often rescaled into fewer gray levels before calculating its GLCMs. However, assuming the more gray levels there are the more information an image contains, we chose 256 gray levels for our transformed image dataset. Research has found that the classification ability of some texture indices decreases when the number of gray levels increases^22^. Future studies can investigate the optimal gray level quantization for crop trait early prediction purpose by rescaling images into 128, 64, 32, 16 or 8 gray levels and comparing the predictions results. Optimal pixel displacement can be explored in a similar manner. Also, instead of only computing GLCMs of two scanning directions, GLCMs of all four scanning directions can be computed and their texture indices can be averaged as more comprehensive representations of a canopy.

A flaw in our image dataset was that the images were not color-calibrated. Image color is subject to the lighting condition, which can cause inconsistent color representations across images, that is, the same pixel value intensity can represent different colors in different images. One common practice for image color calibration is to capture a camera calibration target in all images, such as ColorChecker (X-Rite, Grand Rapids, MI, USA)^44^. Yet, how to effectively implement a calibration target into a high-throughput phenotyping system when measuring thousands of plots remains a challenge for future research.

The cameras employed in this study were not able to capture the fine vasculature of soybean leaves. Vasculature features such as vein density and vein diameter regulate plant mechanical strength and serve as channels for transporting nutrients such as water and minerals^41^, therefore they are crucial for plant photosynthesis. For an image with a sufficient spatial resolution, texture indices can be good indicators for subtle leaf vasculature difference among plant genotypes.

Aside from the modelling techniques that were compared in this study, other machine learning methods such as deep learning algorithms can be examined in the future as they have been demonstrated to have superior regression performances^45,46^. However, large calibration samples are typically required for the success of using such techniques. Also depending on the dataset, for example when the response predictor relationship is strictly linear, even a linear modelling technique such as PLSR can outperform machine learning techniques since machine learning methods may model for unnecessary noises^37^. When the issue of imbalanced data exist, which was the case in the study, merging categories with small sample sizes can be one way to improve classification accuracy. As 5-point scale scoring is a common practice in plant breeding, results displayed in five classes could be more desirable and informative for breeders and we chose not to merge classes.

The soybean image dataset in this study was collected at central and eastern Nebraska areas during the summer growing season of 2016. Without images collected from another location with different environmental conditions or from another year as reference, significant location and year effects on plant end-season performance might exist. Thus, all conclusions made in this article are solely valid for soybean plots growing at central and eastern Nebraska in 2016 and should not be generalized. As the concept of this study is rudimentary, experiments for various crops under diverse environments across multiple years are needed to confirm the validity and applicability of crop trait early prediction through RGB imagery.

## Conclusion

Based on the results of this study, here are a few conclusions that are only valid for soybean growing at central and eastern Nebraska in 2016:

1. For the purpose of soybean trait early prediction through color and texture features of canopy RGB imagery, among the 10 compared modelling techniques, CB was the best regression technique, and RF was the best classification technique.
2. Using both color and texture indices as well as variables that account for soybean plot location difference and data collection timing difference could provide the best prediction results.
3. Theoretical and empirical transformations of RGB images did contain additional color and texture information that could bring marginal improvements to the prediction results.
4. Yield, Maturity and Seed Size were the soybean traits that might be predictable using color and texture features of early-season canopy RGB images.

## Supplementary information

Supplementary Information

## Data Availability

Data of the study are available to readers upon request.
