# Supplementary material for: Early Prediction of Soybean Traits through Color and Texture Features of Canopy RGB Imagery
Source: Sci Rep. 2019 Oct 1;9:14089. doi: 10.1038/s41598-019-50480-x (PMC6773688; doi:10.1038/s41598-019-50480-x)
Supplement: Supplementary file 1 — Supplementary Information [file 41598_2019_50480_MOESM1_ESM.pdf]

Early Prediction of Soybean Traits through Color and Texture Features of Canopy RGB Imagery

Wenan Yuan[1,*], Nuwan Kumara Wijewardane[1], Shawn Jenkins[2], Geng Bai[1], Yufeng Ge[1], and George L. Graef[2]

[1]Biological Systems Engineering Department, University of Nebraska–Lincoln, Lincoln, NE 68583, USA
[2]Department of Agronomy and Horticulture, University of Nebraska–Lincoln, Lincoln, NE 68583, USA
[*]Correspondence: wenan.yuan@huskers.unl.edu

Supplementary Information

Relationships between nine soybean traits:

	**Yield**	**Maturity**	**Lodging**	**Height**	**Seed Size**	**Seed Quality**	**Protein**	**Oil**	**Fiber**
**Yield**	1.0000	0.5286	-0.5996	0.1742	0.5586	0.0189	-0.6771	0.6385	0.3586
**Maturity**	0.5286	1.0000	-0.3766	0.2818	0.3024	0.0134	-0.5154	0.4689	0.2286
**Lodging**	-0.5996	-0.3766	1.0000	0.0728	-0.3982	0.0117	0.6465	-0.6587	-0.2864
**Height**	0.1742	0.2818	0.0728	1.0000	0.1623	-0.0345	0.0070	-0.0169	-0.0168
**Seed Size**	0.5586	0.3024	-0.3982	0.1623	1.0000	-0.1264	-0.3705	0.4906	0.1924
**Seed Quality**	0.0189	0.0134	0.0117	-0.0345	-0.1264	1.0000	-0.0811	0.0309	0.0325
**Protein**	-0.6771	-0.5154	0.6465	0.0070	-0.3705	-0.0811	1.0000	-0.7526	-0.6818
**Oil**	0.6385	0.4689	-0.6587	-0.0169	0.4906	0.0309	-0.7526	1.0000	0.1780
**Fiber**	0.3586	0.2286	-0.2864	-0.0168	0.1924	0.0325	-0.6818	0.1780	1.0000

Table S1. Pearson correlation coefficients between nine soybean traits.

	**Yield**	**Maturity**	**Lodging**	**Height**	**Seed Size**	**Seed Quality**	**Protein**	**Oil**	**Fiber**
**Yield**	1.0000	0.0000	0.0000	0.0000	0.0000	0.4140	0.0000	0.0000	0.0000
**Maturity**	0.0000	1.0000	0.0000	0.0000	0.0000	0.6155	0.0000	0.0000	0.0000
**Lodging**	0.0000	0.0000	1.0000	0.0000	0.0000	0.6610	0.0000	0.0000	0.0000
**Height**	0.0000	0.0000	0.0000	1.0000	0.0000	0.3116	0.7639	0.4689	0.4719
**Seed Size**	0.0000	0.0000	0.0000	0.0000	1.0000	0.0000	0.0000	0.0000	0.0000
**Seed Quality**	0.4140	0.6155	0.6610	0.3116	0.0000	1.0000	0.0008	0.2005	0.1776
**Protein**	0.0000	0.0000	0.0000	0.7639	0.0000	0.0008	1.0000	0.0000	0.0000
**Oil**	0.0000	0.0000	0.0000	0.4689	0.0000	0.2005	0.0000	1.0000	0.0000
**Fiber**	0.0000	0.0000	0.0000	0.4719	0.0000	0.1776	0.0000	0.0000	1.0000

Table S2. P-values for the Pearson correlation coefficients between nine soybean traits.

Full Modelling Results:

Variable	Ground Truth	Technique	RMSE	R^2	Bias
All color + all texture	Yield	CB	10.1416	0.6606	0.5345
		SVR	10.5232	0.6322	0.4999
		RF	10.5238	0.6351	-0.1140
		PLS	11.3891	0.5682	0.0822
		ANN	12.6273	0.4723	-0.1828
	Maturity	CB	3.9240	0.7266	0.3698
		RF	4.0738	0.7076	0.1830
		SVR	4.1821	0.6869	0.3953
		PLS	4.6371	0.6127	0.2775
		ANN	5.5609	0.4446	0.4283
	Height	CB	5.3108	0.2481	-0.0597
		RF	5.3725	0.2117	0.0692
		SVR	5.3852	0.2066	-0.1021
		PLS	5.4230	0.1959	0.0196
		ANN	5.7584	0.0936	0.1102
	Seed Size	CB	1.6322	0.5350	0.0160
		RF	1.7035	0.4798	0.0614
		SVR	1.7292	0.4591	0.0061
		PLS	1.7856	0.4210	0.0349
		ANN	1.8395	0.3870	0.1040
	Protein	CB	1.1367	0.5442	0.0058
		SVR	1.1431	0.5397	-0.0131
		RF	1.1510	0.5388	0.0508
		PLS	1.2173	0.4847	0.0483
		ANN	1.3133	0.4248	0.0523
	Oil	CB	0.7399	0.5996	0.0006
		SVR	0.7444	0.5948	0.0098
		RF	0.7506	0.5920	-0.0604
		ANN	0.7721	0.5797	-0.0596
		PLS	0.7878	0.5512	-0.0519
	Fiber	SVR	0.1723	0.1226	0.0135
		RF	0.1741	0.1178	-0.0073
		CB	0.1742	0.1086	0.0148
		PLS	0.1760	0.0928	-0.0036
		ANN	0.1809	0.0874	-0.0011

Table S3. Regression results for Objective 1 sorted by RMSE.

Variable	Ground Truth	Technique	Accuracy	Kappa
All color + all texture	Lodging	RF	0.7633	0.3682
		PLSDA	0.7479	0.2532
		LDA	0.7465	0.4085
		ANN	0.7149	0.0000
		SVM	0.6791	0.2817
	Seed Quality	RF	0.6555	0.1442
		ANN	0.6443	-0.0076
		PLSDA	0.6425	0.0100
		LDA	0.6127	0.1493
		SVM	0.5922	0.1351

Table S4. Classification results for Objective 1 sorted by Accuracy.

Technique	Ground Truth	Variable	RMSE	R^2	Bias
CB	Yield	All color + all texture + LnT	9.8226	0.6794	0.3044
		All color + all texture	10.1416	0.6606	0.5345
		All texture	10.1779	0.6572	0.4523
		All color	10.8738	0.6067	0.2673
	Maturity	All color + all texture + LnT	3.6995	0.7562	0.2875
		All color + all texture	3.9240	0.7266	0.3698
		All texture	3.9901	0.7177	0.4013
		All color	4.1446	0.6980	0.4113
	Height	All color + all texture + LnT	5.2125	0.2742	-0.0817
		All texture	5.3022	0.2360	-0.0064
		All color + all texture	5.3108	0.2481	-0.0597
		All color	5.6028	0.1590	-0.1313
	Seed Size	All texture	1.6117	0.5386	-0.0007
		All color + all texture + LnT	1.6312	0.5250	-0.0390
		All color + all texture	1.6322	0.5350	0.0160
		All color	1.7237	0.4827	0.0560
	Protein	All color + all texture + LnT	1.1034	0.5710	0.0008
		All texture	1.1147	0.5613	-0.0002
		All color + all texture	1.1367	0.5442	0.0058
		All color	1.1733	0.5141	-0.0211
	Oil	All color + all texture + LnT	0.7205	0.6202	0.0041
		All texture	0.7256	0.6150	-0.0019
		All color + all texture	0.7399	0.5996	0.0006
		All color	0.7665	0.5701	0.0085
	Fiber	All texture	0.1724	0.1247	0.0127
		All color + all texture + LnT	0.1742	0.1131	0.0138
		All color + all texture	0.1742	0.1086	0.0148
		All color	0.1743	0.1221	0.0143

Table S5. Regression results for Objective 2 sorted by RMSE.

Technique	Ground Truth	Variable	Accuracy	Kappa
RF	Lodging	All color + all texture + LnT	0.7662	0.3910
		All color + all texture	0.7633	0.3682
		All color	0.7609	0.3750
		All texture	0.7605	0.3456
	Seed Quality	All color + all texture + LnT	0.6629	0.1687
		All texture	0.6611	0.1688
		All color + all texture	0.6555	0.1442
		All color	0.6350	0.0840

Table S6. Classification results for Objective 2 sorted by Accuracy.

Technique	Ground Truth	Variable	RMSE	R^2	Bias
CB	Yield	All color + all texture + LnT	9.8226	0.6794	0.3044
		RGB color + RGB texture + LnT	10.5667	0.6292	0.5633
	Maturity	All color + all texture + LnT	3.6995	0.7562	0.2875
		RGB color + RGB texture + LnT	3.8692	0.7313	0.2369
	Height	All color + all texture + LnT	5.2125	0.2742	-0.0817
		RGB color + RGB texture + LnT	5.4168	0.1997	-0.2116
	Seed Size	All color + all texture + LnT	1.6312	0.5250	-0.0390
		RGB color + RGB texture + LnT	1.6654	0.4991	-0.0658
	Protein	All color + all texture + LnT	1.1034	0.5710	0.0008
		RGB color + RGB texture + LnT	1.1192	0.5590	-0.0217
	Oil	All color + all texture + LnT	0.7205	0.6202	0.0041
		RGB color + RGB texture + LnT	0.7386	0.6010	-0.0007
	Fiber	All color + all texture + LnT	0.1742	0.1131	0.0138
		RGB color + RGB texture + LnT	0.1742	0.1077	0.0152

Table S7. Regression results for Objective 3 compared to the results of Objective 2.

Technique	Ground Truth	Variable	Accuracy	Kappa
RF	Lodging	All color + all texture + LnT	0.7662	0.3910
		RGB color + RGB texture + LnT	0.7574	0.3817
	Seed Quality	All color + all texture + LnT	0.6629	0.1687
		RGB color + RGB texture + LnT	0.6387	0.1012

Table S8. Classification results for Objective 3 compared to the results of Objective 2.
